# Real-World Experience of Patient’s Compliance and Clinical Outcomes for Trimodality Treatment for Esophageal Cancer: a Study from a Cancer Center in North-East India

**DOI:** 10.1007/s13193-024-01881-6

**Published:** 2024-01-13

**Authors:** Gaurav Das, P. S. Arun, Partha Sarathi Roy, Gautam Sarma, Jyotiman Nath, Deep Jyoti Kalita, Abhijit Talukdar

**Affiliations:** 1 Department of Surgical Oncology, Dr. B. Borooah Cancer Institute, Room No. 30, AK Azad Road, Gopinath Nagar, Guwahati, Assam 781016 India; 2 Department of Surgical Oncology, Dr. B. Borooah Cancer Institute, AK Azad Road, Gopinath Nagar, Guwahati, Assam 781016 India; 3 Department of Medical Oncology, Dr. B. Borooah Cancer Institute, Room No. 24, AK Azad Road, Gopinath Nagar, Guwahati, Assam 781016 India; 4 Department of Radiation Oncology, Dr. B. Borooah Cancer Institute, Room No. 109, AK Azad Road, Gopinath Nagar, Guwahati, Assam 781016 India; 5 Department of Surgical Oncology, Dr. B. Borooah Cancer Institute, Room No. 27, AK Azad Road, Gopinath Nagar, Guwahati, Assam 781016 India; 6 Department of Surgical Oncology, Dr. B. Borooah Cancer Institute, Room No. 28, AK Azad Road, Gopinath Nagar, Guwahati, Assam 781016 India

**Keywords:** Esophageal cancer, Neoadjuvant chemoradiotherapy, Trimodality treatment

## Abstract

Preoperative chemoradiotherapy is a standard treatment for patients with locally advanced, resectable esophageal cancer. The treatment completion rates impact the survival outcomes (Eyck et al J Clin Oncol 39(18):1995–2004, 2021). Thus, we aimed to estimate the effect of neoadjuvant chemoradiotherapy (NACRT) in terms of treatment completion rates and survival in this subset of patients and bring out the clinical outcomes in that context. This was a retrospective study done at a tertiary cancer center in North-East India. The study period was from 1 January 2018 to 31 December 2021. We included patients diagnosed with locally advanced and resectable esophageal cancer (cT_2-3_N_any_M0) involving the middle and/or lower thoracic esophagus and who were planned for trimodality treatment in the Joint Tumor Board. Out of the 82 patients who were planned for trimodality treatment, all were squamous cell carcinomas. We found that 54.9% of patients completed the entire trimodality treatment. The median age was 56 years (range 34 to 73 years). The male to female ratio was 59:23. Adverse events, of any grade, were seen in 76% of patients who received NACRT. Fatigue (66%) was the most common toxicity. The common hematologic toxicities were neutropenia and anemia (7.3% each). A total of 45 patients (54.9%) were able to complete all the three modalities of treatment. Transthoracic esophagectomy was the preferred approach (84.4%). The site of anastomosis was in the neck of all the patients. Anastomotic leak was seen in 17.7% of patients. Postoperative pulmonary and cardiac complications occurred in 31.1% and 8.9% of patients respectively. The 30-day mortality was 6.7% (three deaths). A pathological complete response was seen in 35.6% among patients who underwent an esophagectomy. R0 resection was achieved in 93.3% of patients. The median overall survival and disease-free survival were 19 months and 17 months respectively. The completion rate of trimodality treatment in the real-world scenario was found to be low in our study, the reasons for which need to be identified and effectively resolved. Oncological outcomes were similar to the published literature.

## Introduction

Esophageal cancer (EC) is the seventh most common cancer worldwide and the sixth most common cause of death [1]. There is a regional variation in its incidence and pathology. India has a high incidence of EC with a higher proportion of squamous cell cancers (SCC). A very high incidence has been reported in the North-East region of India. EC presents in a locally advanced stage in the majority of patients and carries a poor prognosis [2]. Preoperative chemoradiotherapy has become a standard of care for patients with locally advanced resectable esophageal. Treatment completion rates significantly impact the survival rates. There are a variety of factors like disease-related malnutrition and deterioration of performance status due to treatment-related toxicities which compromise the compliance of patients to the treatment protocol. The present study aims to estimate the treatment compliance rate in a high-incidence region. This real-world evidence to understand the magnitude of the problem will enable us to devise strategies to identify the causes and mitigate them.

## Methods

This was a retrospective study done at a tertiary cancer center in North-East India. The study period was from 1 January 2018 to 31 December 2021. We included patients diagnosed with locally advanced and resectable esophageal cancer (cT_2-3_N_any_M0) involving the middle and/or lower thoracic esophagus and who were planned for trimodality treatment in the Joint Tumor Board. Trimodality treatment comprised of neoadjuvant chemoradiation followed by an esophagectomy with nodal dissection. Patients with recurrent or residual cancer who underwent salvage esophagectomy and those having their disease in the upper thoracic esophagus were excluded from the study. The data of all the patients who were planned for NACRT followed by esophagectomy was collected from the Joint Tumor Board register, the radiotherapy treatment register, the surgical procedures register, and the patient’s case records (files and electronic medical records). From this data, the actual number of patients who successfully completed NACRT was found and the adverse events associated with NACRT were noted. The clinical details of all the patients including the history, physical examination, pre-NACRT, and post-NACRT clinical staging based on contrast-enhanced CT scan (CECT) of the neck, thorax, abdomen, pelvis, and upper GI endoscopy were documented. For the purpose of disease staging, CECT scan as stated above was used for all patients. PET CT scan and endoscopic ultrasound (EUS) were not used for disease staging. The study was approved by the institutional ethical committee (IEC).

## Treatment

### Chemoradiation

Two NACRT regimens were found. One followed the CROSS trial which was administered. A total radiation dose of 41.4 Gy was given in 23 fractions of 1.8 Gy each, with 5 fractions administered per week, starting on the first day of the first chemotherapy cycle. Carboplatin targeted an area under the curve of 2 mg per milliliter per minute, and paclitaxel at a dose of 50 mg per square meter of body-surface area was administered intravenously along with radiation on days 1, 8, 15, 22, and 29.

The second protocol followed was 50.4 Gy along with cisplatin (100 mg/m^2^/day on day 1) and fluorouracil (1000 mg/m^2^/day on days 1 to 4) every 4 weeks for two cycles.

All the patients had their post-NACRT imaging and upper GI endoscopy done at 3 to 5 weeks from the completion of NACRT. The number of patients who defaulted after NACRT was noted. The toxicity associated with chemoradiation was graded by using Common Terminology Criteria for Adverse Events version 5.0 (v5.0 CTCAE) of the National Cancer Institute (NCI).

### Surgery

The final number of patients who subsequently underwent esophagectomy was considered for further documentation. The type of surgery (open vs minimally invasive) (transthoracic vs transhiatal approach) and intraoperative details including the type of nodal dissection were noted. Postoperative morbidities along with the duration of ICU stay, hospital stay, and postoperative day of discharge were recorded.

### Pathological Analysis

The histopathological examination findings including tumor type and length of the segment involved, lymph node yield and number of positive lymph nodes, resection margins, pathological complete response (PCR), and R0 resection of all the patients were noted. If a tumor was present at 1 mm or less from the proximal, distal, or circumferential resection margin, it was microscopically positive (R1).

### Statistical Analysis

The archived case records of the patients were studied and presented in the form of median values, ranges, ratios, and percentages. All data collected from January 2018 to December 2021 were included in the analysis, with a follow-up period range of 1 to 4 years. In case a disease recurrence has happened in follow-up, the time of recurrence was noted and the disease-free period after completion of trimodality treatment was calculated. In case the patient defaulted the follow-up, the last follow-up date was considered the event and the survival analysis was done. The Kaplan–Meier method was used to estimate survival. The association between the median overall survival time and the different independent parameters like age, gender, body mass index, location, grade, number of days of ICU stay, anastomotic leak, pCR, and number of positive lymph nodes was done by performing the chi-square test for independent of attributes.

## Results

**Fig. 1:**
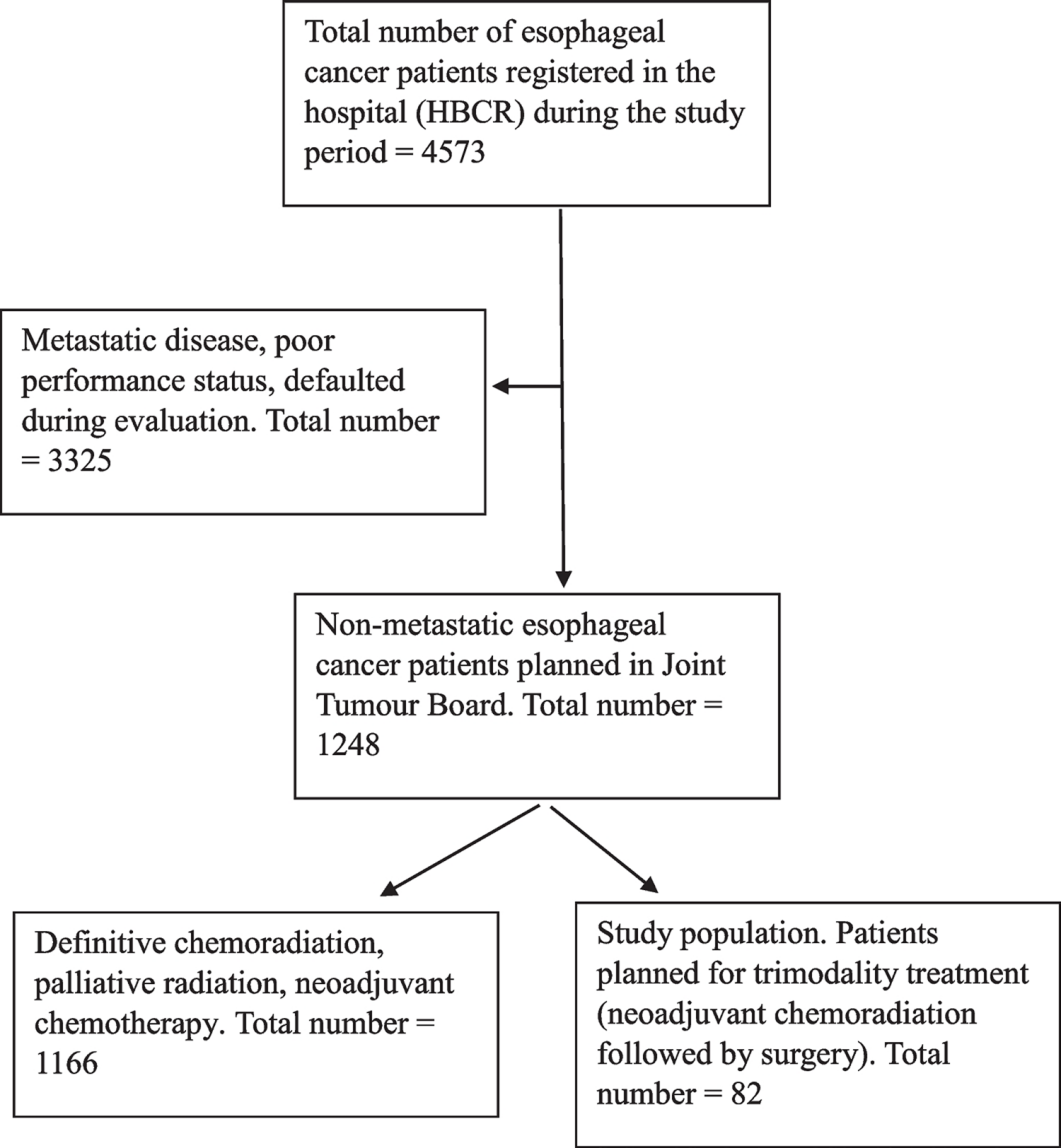
Patient selection algorithm

A total of 82 patients with locally advanced esophageal cancer were planned for trimodality treatment as per the decision taken in the Joint Tumor Board meeting. The patient selection algorithm has been shown in Fig. 1. Out of 82 patients, only 58 patients completed NACRT (70.7%). Nine patients among those who completed NACRT defaulted before further treatment. Forty-nine patients were evaluated further and planned for surgery. Out of these, four patients were found inoperable during surgery. Among the four patients who were found to be inoperable during surgery, three were operated on after 8 weeks of neoadjuvant chemoradiation (NACRT) and one patient was operated on 6 weeks after NACRT. Only 45 patients completed the scheduled trimodality treatment (54.9%) (Table 1).

**Table 1:** The trimodality treatment compliance

Treatment planned/received	No of patients (percentage)
Total no. of patients planned for NACRT (JTB)	**82**
Completed NACRT	**58 (70.7%)**
Defaulted after NACRT^*^	**9 (15.5%)**
Inoperable disease during surgery^**^	**4 (4.9%)**
Completed trimodality treatment	**45 (54.9%)**
Surgery done at 4 to 6 weeks	**20**
Surgery done at 6 to 8 weeks	**18**
Surgery done at 8 to 12 weeks	**5**
Surgery done after 12 weeks but within 6 months^***^	**2**

NACRT, neoadjuvant chemoradiation

JTB, Joint Tumor Board

^*^Patients defaulted for further treatment due to personal reasons and/or toxicity due to NACRT

^**^Resection was not possible because the primary tumor or lymph nodes were identified as unresectable during surgery

^***^One patient had prolonged morbidity after NACRT and got operated on after 16 weeks and one more patient defaulted and came for a follow-up after 14 weeks and underwent surgery

The median age of the patients was 56 years (range 34 to 73 years). The male to female ratio was 59:23. Thirty-nine patients (47.6%) had their tumor epicenter located in the middle thoracic esophagus and 43 patients (52.4%) had it located in the lower third. The histological subtype found among all the patients was squamous cell carcinoma (100%) with different histologic grades. Fifty-five patients had enlarged nodes in pretreatment imaging (67.1%) (Table 2).

**Table 2:** Patient and disease characteristics

Characteristics	Numbers (percentage)
Median age	56 years (34 to 73 years)
Male to female ratio	59: 23
Median BMI	19.1 (17.3 to 23.)
Smoking history	62 (75.6%)
Tumor location among all patients	
Middle thoracic	39 (47.6%)
Lower thoracic	43 (52.4%)
Tumor location among operated patients	
Mid thoracic only (M)	15
Lower thoracic (L)	15
Mid thoracic and lower thoracic (ML)	10
Lower thoracic involving gastroesophageal junction (GEJ)	5
Histological subtype	
Squamous cell carcinoma (SCC)	82 (100%)
Well differentiated SCC	17 (20.7%)
Moderately differentiated SCC	45 (54.8%)
Poorly differentiated SCC	20 (24.5%)
cN + at inclusion	55 (67.1%)

*BMI*, body mass index

*SCC*, squamous cell carcinoma

*cN* + , clinically node positive based on CT scan

Thirty-two patients (76%) had some form of adverse events of any grade during and after NACRT. Fatigue was the most common toxicity noticed in 28 patients (66%). The most common major hematologic toxicity encountered was neutropenia and anemia (7.3% each). Three patients required intervention for neutropenia. Nausea was the most common gastrointestinal toxicity noticed in 16 patients (38%) (Table 3).

**Table 3:** Adverse events during neoadjuvant chemoradiation

Event	Number of patients	Grades 1, 2	Grade 3	Grades 4, 5
Nausea	16	14	2	NA
Vomiting	8	4	3	0
Diarrhea	6	3	2	0
Constipation	2	2	0	0
Fatigue	28	24	4	NA
Weight loss	4	4	0	NA
Neutropenia	6	3	2	1
Thrombocytopenia	5	3	1	1
Anemia	6	4	2	0
Neurotoxic side effects	4	3	1	0
Dermatitis	3	3	0	0
Alopecia	5	5	NA	NA

*NA* not applicable because there is no such grade in the event mentioned as per CTCAE v5.0

Among 45 patients who underwent esophagectomy, 38 patients (84.4%) had it done by transthoracic approach and 7 patients had it by transhiatal approach. Five patients out of 38 patients who underwent transthoracic surgery had open surgery and the remaining 33 patients had VATS (video-assisted thoracoscopic surgery). There were two conversions from VATS to open surgery. All the seven patients who underwent transhiatal approach had a lower thoracic cancer and it was purely the decision of the operating surgeon to choose that approach. As the surgical unit comprised of several operating surgeons, preferences for surgical approaches were varied, depending on tumor and patient characteristics. The indication for conversion from VATS to open surgery in the two patients mentioned in the study was intraoperative bleeding (Table 4).

**Table 4:** Types of surgeries done

Types of esophagectomies done	Numbers (percentage)
Transthoracic esophagectomies	38 (84.4%)
Transhiatal; esophagectomies	7 (16.6%)
Open TTE	5
Open McKeown’s TTE	5
With 2-field lymphadenectomy	4
With extended 2-field lymphadenectomy	1
With 3-field lymphadenectomies	0
Open Ivor Lewis TTE	0
VATS TTE	30
With 2-field lymphadenectomies	14
With extended 2-field lymphadenectomy	15
With 3-field lymphadenectomies	1
VATS converted to open TTE	2

The site of anastomosis was the neck in all 45 patients. Stapled anastomosis and hand-sewn anastomosis were done in 42 patients and 3 patients respectively. Postoperative complications (all Clavien-Dindo grades) happened in 20 patients (44.4%). Anastomotic leak occurred in eight patients (17.7%). All the patients were managed conservatively. All these leaks required bedside opening of the neck wound and regular dressing. By definitions of Esophagectomy Complications Consensus Group (ECCG), these were all type II leaks [3]. With the above measures, the leaks healed during the course of the same hospital admission in five patients before the discharge of the patient, and the leak output decreased substantially in three patients before the discharge of the patient and was healed subsequently at a follow-up visit to the hospital. Hoarseness of voice was noticed in seven patients but none of them had a documented vocal cord palsy. Pulmonary complications happened in nine patients (21%). Surgical site infection (SSI) was seen in nine patients and all of them had a superficial SSI which was settled with dressing and appropriate antibiotics. Postoperative in-hospital mortality happened in three patients. The cause of death was pneumonia and sepsis in two patients and myocardial infarction in one patient (Table 5).

**Table 5:** Postoperative complications

Postoperative events	Number of patients (percentage)
Postop mechanical ventilation	26 (57.7)
Pulmonary complications	14 (31.1)
Cardiac complications	4 (8.9)
Anastomotic leakage	8 (17.8)
SSI	9 (20)
Chyle leakage	2 (4.4)
Death in hospital	3 (6.7)
ICU stay beyond 3 days	12 (26.7)

### Pathological Assessment

R0 resection was possible in 42 out of 45 patients (93.3%). A pathologic complete response (pCR) was seen in 16 patients among patients who underwent an esophagectomy (35.6%). The pathological complete response (pCR) rate was 31.8% with CROSS regimen treatment (14 out of 44) and it was 40% with the CF and radiation treatment (2 out of 5). It is noteworthy that the CROSS regimen was the predominantly used one (89%). The overall pCR rate was 32.7% (16 out of 49, including the four inoperable cases) and 35.6% among those who underwent an esophagectomy. We have looked into the pathological response rates with respect to the tumor location. Among the patients with disease in the middle third of the thoracic esophagus, the pCR rate was 46.7% (7 out of 15); in those with disease in the lower third, the pCR rate was 33.3% (5 out of 15); in those with disease in the lower thoracic esophagus with extension into gastroesophageal junction (GEJ), the pCR rate was 40% (2 out of 5); and finally, in those who had long segment disease from middle to lower thoracic esophagus, the pCR rate was 20% (2 out of 10).

The median lymph nodal yield was 13 (range, 3 to 31). The range of nodal yield was 3 to 31 lymph nodes. A nodal yield of ≤ 12 nodes was seen in 55.6% of patients and a nodal yield of > 12 nodes was seen in the remaining 44.4% of patients. The range of positive lymph nodes was 0 to 6 and the range of metastatic lymph node ratio (number of positive nodes divided by number of nodes harvested) was 0 to 0.46. Perineural invasion was noticed in four patients and lymphovascular emboli were noted in three patients (Table 6).

**Table 6:** Pathological outcomes

Factor	Number (percentage)
Margin status	
R0	42 (93.3)
R1 or R2	3 (6.7)
pCR	16 (35.6)
Nodal yield	
≤ 12	25 (55.6)
> 12	20 (44.4)
Number of positive nodes	
≤ 2	41 (91.1)
> 2	4 (8.9)
Perineural invasion	4 (8.9)
Lymphovascular emboli	3 (6.7)

*pCR*, pathologic complete response

*R0*, microscopic negative margins (i.e., no tumor within 1 mm of the resection margins)

*R1*, microscopic positive margins (i.e., tumor cells found within 1 mm of the resection margins)

*R2*, macroscopic positive margins

### Follow-up and Survival

**Fig. 2:**
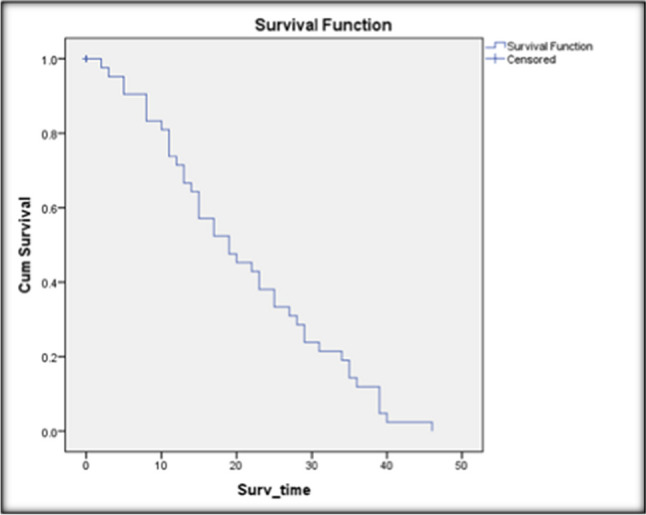
Survival curve for overall survival for trimodality treatment

The median follow-up for the surviving patients was 16 months. The overall survival time ranged between 11.6 and 26.4 months. The median overall survival time was found to be 19 months (Table 7 and 8, Fig. 2).

**Table 7:** Summary of overall survival

Total no. of cases	No. of cases experienced the event	No. of censored cases
45	42	3

**Table 8:** Median survival time for overall survival

Median survival time (in months)	Standard error	95% confidence interval	
		Lower bound	Upper bound
19	3.776	11.599	26.401

**Fig. 3:**
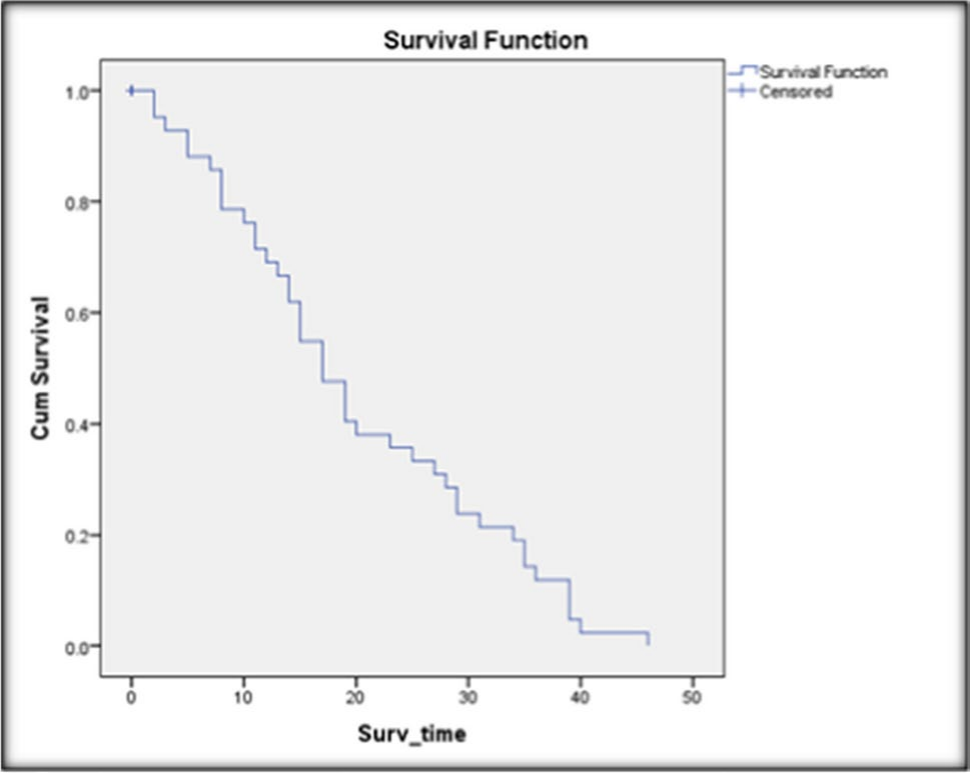
Survival curve for disease-free survival for trimodality treatment

Disease recurrence was noted in 8 out of 42 patients who were on follow-up (19%). Loco-regional recurrence occurred in five patients and two patients had a distant recurrence in the brain and liver respectively. One patient developed both loco-regional and distant disease recurrence. The disease-free survival of surviving patients ranged between 13.5 and 20.5 months. The median disease-free survival (DFS) was found to be 17 months (Table 9 and 10, Fig. 3).

**Table 9:** Disease-free survival

Total no. of cases	No. of cases with an event	No. of censored cases
45	42	3

**Table 10:** Median survival time for disease-free survival

Median survival time (in months)	Standard error	95% confidence interval	
		Lower bound	Upper bound
17	1.798	13.476	20.524

We were interested in looking into the association between the median overall survival time and the different independent parameters and thus performed the chi-square test for independent attributes. The associations between the median overall survival time and the different independent parameters like age, gender, BMI, location, grade, number of days of ICU stay, anastomotic leak, pCR, and number of positive lymph nodes were not statistically significant. The *p*-values of none of the parameters were found to be less than 0.05. Hence, the null hypothesis was accepted indicating that none of the parameters had a statistically significant association with overall survival (Table 11).

**Table 11:** Result of associations between the median overall survival time and the different independent parameters

Parameter	Median OS		*p*-value
	≤ 19 months	> 19 months	
Age group			
≤ 55	15	10	0.502
> 55	10	10	
Gender			
Male	19	11	0.138
Female	06	09	
BMI group			
≤ 20	09	09	0.540
> 20	16	11	
Location_group			
L	07	08	0.675
LJ	04	01	
M	08	07	
ML	06	04	
Grade			
Grade I	05	04	0.267
Grade II	19	12	
Grade III	01	04	
Staying in ICU			
≤ 3 Days	13	16	0.051
> 3 Days	12	04	
Anastomotic leak			
Yes	04	06	0.262
No	21	14	
PCR group			
Yes	10	06	0.486
No	15	14	
No. of positive LN			
≤ 2	23	18	0.815
> 2	02	02	

## Discussion

Neoadjuvant chemoradiation (NACRT) is one of the standard preoperative treatment strategies for locally advanced and resectable esophageal cancer, the other being neoadjuvant chemotherapy (NACT). NACRT or NACT increases the 5-year overall survival of patients by about 10% [4, 5]. However, there is no convincing evidence favoring one approach over the other, and so, different institutions have different preferences regarding which one to use. Our center adopted the practice of neoadjuvant chemoradiation in the year 2018, and it is increasingly being preferred over NACT, which was the predominant strategy prior to that period. We have actually studied the population of esophageal cancers who were intended to be treated with trimodality treatment (neoadjuvant chemoradiation followed by esophagectomy). In our institute, the predominant histology of esophageal cancer is squamous cell carcinoma and the adenocarcinomas are mostly seen to present as gastroesophageal junction cancers. The treatment usually offered to the latter subgroup is neoadjuvant chemotherapy followed by surgery. This is the reason as to why the squamous cell carcinoma histology has been found to be 100% in our study. NACRT has the potential to downstage the disease, increase the rate of R0 resections, eradicate occult micrometastasis, and reduce the local recurrence. The concurrent use of radiation (41.4 Gy in 23 fractions) with five cycles of paclitaxel and carboplatin has become one of the standards of care after the landmark trial by van Hagen et al. High pCR rates are seen for the subset of patients with squamous cell carcinoma compared to adenocarcinoma [6]. The same protocol had been applied to the majority of our patients (89%). The remaining patients received a different protocol as mentioned in the methodology section.

Our retrospective analysis revealed a treatment completion rate of only 54.9% with the trimodality treatment. This excludes the four patients (4.9%) who were found to have inoperable disease at the time of surgery. The reasons behind such a suboptimal compliance could not be assessed properly because of the retrospective nature of the study. This percentage is very low when compared with the other prospective and retrospective trials conducted for NACRT followed by surgery. We have identified the problem area with this retrospective study and we have to prospectively identify the real-world reasons for it. In the CROSS trial, out of 180 patients randomized to the neoadjuvant chemoradiation arm, 161 patients underwent an esophagectomy (89.4%) [7]. In the NEOCTREC_50_10 randomized controlled trial, 82.6% of patients completed the trimodality treatment [8]. In a prospective single-arm study from a tertiary cancer center in India, out of 50 patients, 32 patients eventually underwent an esophagectomy after neoadjuvant chemoradiation, translating to a 64% treatment completion rate [8]. Another tertiary cancer center from the Eastern region of India reported a 73.7% treatment completion rate in the real-world scenario after applying the CROSS protocol [9]. The POWERRANGER trial is a randomized controlled trial comparing preoperative chemotherapy with chemoradiation for esophageal and gastroesophageal junction cancers and is looking into treatment compliance as a primary endpoint [10].

Studies which have evaluated the efficacy of the CROSS regimen in the real-world scenario found that if recruitment of patients is beyond the criteria stipulated in that trial, then outcomes like pathological complete response (pCR), median overall survival, and postoperative morbidity and mortality were inferior [11, 12]. A propensity-score matched study showed that cisplatin/5-fluorouracil combination with radiotherapy had similar results compared to paclitaxel/carboplatin combination with radiotherapy, as was used in the CROSS trial [13]. Our patients received CROSS protocol treatment in 89% of cases, and the remaining patients received a cisplatin/5-fluorouracil combination with radiation. All patients who underwent an esophagectomy after NACRT were followed up. The use of adjuvant immunotherapy was not seen in any of the patients. The results of the CHECKMATE 577 trial which was published in April 2021 showed a significant disease-free survival advantage in patients who received adjuvant nivolumab treatment [14]. However, this practice had not been adopted till the end of the study period of our study (Dec 2021).

The median disease-free survival was 17 months and the median overall survival was 19 months. The analysis of the association of factors like age, gender, body mass index, location, grade, number of days of ICU stay, anastomotic leak, pCR, and number of positive lymph nodes did not show a statistically significant association with overall survival. This may be attributed to a relatively small sample size of only 82 patients, which is a limitation of the study.

Complete remission of both the primary tumor and the lymph nodes (ypT0N0) was the most favorable pathological outcome of chemoradiotherapy. Pathological complete response of 35.6% observed in our study was in line with reported percentages in other prospective studies. A high percentage of R0 resections (93.3%) was probably the reflection of a high proportion of downstaging as a result of chemoradiation which correlates with the values of randomized controlled trials. Both these figures were expectedly high as 100% of patients were squamous cell carcinoma, which has better response rates compared to adenocarcinoma [15].

## Conclusion

The compliance rate for trimodality approach is one key area for improvement in esophageal cancer treatment. NACRT is associated with pCR in up to a third of patients and ensures high R0 resection rates. Further prospective studies are needed to assess the real-world reasons for poor compliance and effectively mitigate them.

## Limitations

The retrospective nature of the study is a limitation. Another one is that chemotherapeutic agents and dosage of radiation were not uniform, though 88.9% of patients received NACRT as per the same protocol. The sample size of 82 patients was a drawback as it is thought to have been the reason for the lack of significant association in the statistical analysis of the study variables. This is by virtue of this being an initial experience, as NACRT was started in 2018 in the study center.

## Data Availability

Data is available with the corresponding author.
